# The relationship between death distress and fertility intentions in married women after the earthquake

**DOI:** 10.1017/S1463423626101315

**Published:** 2026-06-22

**Authors:** Serap BATI, Sibel KIYAK

**Affiliations:** Seydisehir Kamil Akkanat Faculty of Health Sciences, Necmettin Erbakan University, Türkiye

**Keywords:** death anxiety, death depression, death distress, disaster, fertility intention

## Abstract

**Objectives:**

Earthquake is a traumatic event with significant physiological and psychological effects, profoundly altering individuals’ lives. This study aimed to determine the relationship between death distress and fertility intentions among women who experienced the Kahramanmaras earthquake in Türkiye on February 6, 2023.

**Methods:**

This descriptive, correlational study included 294 married women residing in an earthquake-affected region of Turkey. We collected data using the Participant Information Form and the Brief Death Distress Scale.

**Results:**

A total of 294 women participated in this study. The mean death distress score was 25.65 ± 7.46. The death depression dimension had the highest score among the subdimensions of the death distress scale (11.02 ± 3.61). Age, income level, pregnancy, number of children, extent of house damage during the earthquake, and experience of being trapped under debris were factors influencing death-related distress. Women who changed their fertility intentions had higher levels of death distress, anxiety, and obsession than those who did not change their fertility intentions.

**Conclusions:**

This study provides a new perspective for understanding post-earthquake changes in married women’s mental health and fertility behavior. Death distress is a key factor that should be considered in planning post-disaster healthcare services for women.

## Introduction

On February 6, 2023, a 7.7 magnitude seismic event centered in the city of Kahramanmaraş, Türkiye, resulted in significant devastation and losses across 11 surrounding provinces, leading to the declaration of the region as a disaster zone (Özmen, 2023). The earthquake caused the loss of 50,500 lives. In the month following the earthquake, 11,020 aftershocks occurred. Regional inspections indicated that 215,000 buildings either collapsed or sustained serious damage as a result of the earthquake. Thousands of people have lost their homes and loved ones (AFAD, 2023).

Earthquakes not only cause physical destruction but also deeply impact on the mental health of individuals in the affected geographical area (Nakajima, 2012). Individuals who have been exposed to such significant traumatic events often experience adverse effects on their mental health (Kartol *et al.,* 2024; Kristensen *et al.,* 2012). After an earthquake, people may experience significant emotions, such as shock, disbelief, anger, helplessness, powerlessness, loss of trust, loss of control, and fear of death (Yıldız and Akkoyun, 2023). Among the emotional effects of earthquakes, concerns about death are particularly important. This may manifest as anxiety about death, thoughts related to death, and death depression (Mohammadzadeh *et al.,* 2018). It was observed that 82% of the individuals who were exposed to an earthquake stated that they often thought about death after the earthquake and 69.7% stated that their priorities about life changed (Şeker and Akman, 2014). Furthermore, individuals directly affected by the earthquake experienced higher levels of death anxiety and death distress than their second-degree relatives who were affected by the earthquake. In the same study, it was found that women experienced a higher level of death distress than men (Bırni *et al*., 2023). These findings indicate that the impact of the earthquake extends beyond physical destruction and includes psychological consequences for individuals.

Significant changes in human life can lead individuals to reconsider life priorities and goals (Malicka *et al*., 2021). These changes include economic, social, humanitarian, and health crises that can often reduce the desire to conceive or cause individuals to postpone the decision to have children (Brée *et al*., 2016; Ayllón, 2019; Marteleto *et al*., 2023; Somefun *et al*., 2024). A systematic review on the impacts of disasters showed that disasters generally have a negative impact on fertility rates, although the effect varies depending on the type of disaster (Lee *et al*., 2023). Fertility intention expresses an individual’s desire to have children and is influenced by biological, socioeconomic, cultural, and religious factors (Lau *et al*., 2018). Fertility intention is an important factor that determines the fertility level of families. Some studies focus on women’s fertility intentions in the short term and examine their desire to have children in the next 3 years (Eurostat, 2020; Mussino *et al*., 2021). This is an important approach to understanding how their fertility planning is affected.

According to the Centers for Disease Control and Prevention (CDC), 34 million people in the world are affected by disasters each year, of whom 80% are women and children. It is estimated that 25% of the affected women are of childbearing age (CDC, 2021). Therefore, disasters do not have the same impact on men and women, but their effects on women of childbearing age are more severe. It has been emphasized in the literature that disasters can impact fertility (Zotti *et al*., 2013). With the increasing frequency and intensity of disasters, understanding the effect of disasters on fertility has become an important research topic (Grace, 2017; Muttarak, 2021). However, the cause of this effect remains unclear, as studies have mainly focused on fertility behavior. Fertility intentions measure future fertility behavior over time, and understanding these intentions is crucial in the planning of services for individuals (Malicka *et al*., 2021).

## Aims & hypotheses

This study aimed to investigate the relationship between death distress and fertility intentions among married women of childbearing age residing in the region affected by the earthquake of February 6, 2023. The research question was as follows: i) What is the death distress level among married women residing in the earthquake-affected region following the earthquake on February 6, 2023? ii) What is the relationship between women’s death-distress levels and descriptive characteristics? iii) What is the status of changes in women’s fertility intentions after the earthquake? iv) Is there a relationship between women’s fertility intentions and death distress levels?

## Materials and methods

### Type of research

This study is descriptive and correlational.

### Location and characteristics of the research

The study was conducted in 11 provinces that were affected by the Kahramanmaraş-centered earthquake on February 6, 2023, and were declared disaster zones (Kahramanmaraş, Adana, Adıyaman, Diyarbakir, Gaziantep, Hatay, Kilis, Malatya, Osmaniye, Şanlıurfa, and Elazig).

### Population and sample

The study population consisted of married women aged 18–49 years who resided in the provinces affected by the earthquake centered in Kahramanmaraş on February 6, 2023, and who continued to reside in these provinces after they were declared disaster zones following the earthquake. The study sample consisted of 294 women who met the inclusion criteria and agreed to participate.

G*Power version 3.1.9.2 was used to calculate the sample size (Faul *et al*., 2009). Birni et al., reported the mean death anxiety scores of the participants, which were found to be 25.8 4 ± .34. Using these data, the required sample size was determined to be 265 individuals for a 95% confidence level (1−*α*), 80% power (1−*β*), an effect size of *d* = .176, and a two-tailed *t*-test. To account for potential data loss, the sample size was increased by 10%, resulting in a final sample size of 292 individuals.

### Inclusion criteria for participants

Women who were residing in the provinces declared as disaster zones during the earthquake centered in Kahramanmaraş and Elbistan on February 6, 2023, and who continued to live in the same province after the earthquake, aged 18–49 years, and had been married for more than one year.

### Exclusion criteria

Women with diagnosed or reported psychological problems (depression, suicidal tendencies, etc.), who reported having experienced a traumatic event in the last year before the earthquake, and who had undergone tubal ligation before the earthquake.

### Data collection techniques and tools

Data were collected using the ‘Participant Information Form’ and the ‘Brief Death Distress Scale’.

#### Participant information form

This form was created by the researcher and included questions that inquire about the socio-demographic and obstetric characteristics, experiences about the earthquake, and fertility intentions of the participants (Luppi *et al*., 2020; Bırni *et al*., 2023).

**Figure 1.**
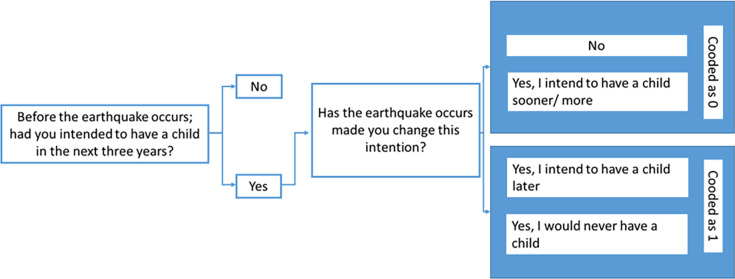
Figure 1 long description. Variable interpretation ploot.

One of the primary objectives of the study was to determine changes in fertility intentions. In this regard, the literature contains similar studies with comparable measurements (Luppi *et al*., 2020; Malicka *et al*., 2021). To measure fertility intention, women were asked, ‘Before the earthquake; were you planning to have children within the next 3 years?’ Those who answered yes to this question were asked the follow-up question, ‘Did the earthquake change your intention to have children?’ and prompted to choose one of the following options: ‘(a) No, (b) Yes, I plan to have children earlier/I plan to have more children, (c) Yes, I postponed my intention to have children until later, (d) Yes, I want to have fewer or no children compared to before the earthquake’. Since only a small number of participants (*n* = 3) stated that they planned to have children earlier or to have more children, participants were divided into two categories and coded as ‘No change, Earlier/More’ (0) and ‘Postponing, Giving up’ (1) (Figure 1).

#### Brief Death Distress Scale (DDS)

The scale was developed by Dadfar and Lerter (2020) to measure death distress. It consists of nine items. The scale has three sub-dimensions: ‘Death Anxiety’ (items 1, 2, and 3), ‘Death Depression’ (items 4, 5, and 6), and ‘Death Obsession’ (items 7, 8, and 9). Each item is rated on a five-point Likert scale (1 = never, 5 = always). There are no reverse-coded items in the evaluation of the scale. The total score is obtained by summing the Likert values of the participant’s responses. The lowest possible score on the scale is nine, and the highest is forty-five. The adaptation of the scale to Turkish was performed by Yıldırım & Güler (2021), and the internal consistency coefficients were calculated as .77 for death anxiety, .88 for death depression, and .91 for death obsession. In the present study, the internal consistency coefficient for the total scale was .80, and the coefficients for the sub-dimensions were .80 for death anxiety, .73 for death depression, and .90 for death obsession.

### Data collection

The data for the study were collected using an online survey. The survey was created using Google Forms and distributed to participants via social media platforms (WhatsApp, Facebook, etc.). Prior the study, a pilot study was conducted with 10 participants who had characteristics similar to those of the sample to assess the comprehensibility of the survey form.

### Statistical analysis

Data were analyzed using SPSS (Statistical Package for the Social Sciences) version 22.0. The conformity of the variables to normal distribution was assessed based on Skewness and Kurtosis values within the range of +1 and −1 (Leech, 2007). Accordingly, the scores for the scale and its sub-dimensions used in the study were normally distributed. To assess the reliability of the measurement obtained from the scale, the internal consistency coefficient (Cronbach’s alpha) was calculated. Descriptive statistics (means, standard deviations, frequencies, and percentages) and analytical statistics (one-way analysis of variance, independent *t*-test, and Spearman’s correlation) were used to analyze the data. *p* < .05 was considered statistically significant in all analyses.

## Results

Table 1 presents descriptive data on women’s demographic characteristics, earthquake experiences, fertility characteristics, and fertility intentions.

**Table 1.** Descriptive data on women’s demographics, earthquake experience, fertility characteristics, the explained change in childbearing intention due to the Earthquake Table 1 long description.

	*n*	%
**Age (mean ± sd)**	32.97 **±** 6.68	Min: 19 – Max: 48
**Number of pregnancies (mean±sd)**	2.65 ± 1.7	Min: 0 Max: 9
**Number of children (mean±sd)**	2.27 ± 1.36	Min: 0 Max: 6
**Education**		
Primary education	92	31.1
High school	117	39.5
University and further	87	29.4
**Working**		
Working	68	23.0
Not working	228	77.0
**Income**		
Income-less than expenses	104	35.1
Income equals expenses	134	45.3
Income exceeds expenses	58	19.6
**Damage to houses lived in due to earthquake**		
No damage occurred	62	20.9
Minor damage occurred	112	37.8
Moderate damage occurred	54	18.2
High level of damage occurred	68	23.1
**Situation under rubble in an earthquake**		
Yes	18	6.1
No	278	93.9
**Injury in earthquake**		
Yes	49	16.6
No	247	83.4
**Experiencing loss due to earthquake**		
No	125	42.2
Yes	171	57.8
Husband/child	5	1.7
Relatives	115	38.9
Friends	51	17.2
**The family planning method he used before the earthquake**		
Not using method	79	26.7
Traditional method	62	20.9
Modern method	155	52.4
**Family planning method currently used**		
Not using method	88	29.7
Traditional method	56	18.9
Modern method	152	51.4
**Fertility intention**		
***Original sample size***	**296**	–
Not intending to have a child before the earthquake	191	64.5
***Sample of those intending to have a child before the earthquake***	**105**	35.5
No change in intention	41	28.57
Intention to have a child sooner/more	3	2.86
Intention to have a child later	30	29.52
**Total**	**296**	**100.0**

Table 2 presents variation in death distress across demographic characteristics, earthquake experience, fertility intentions, and changes in fertility intentions. Those with income higher than their expenses had lower death distress compared with those with income equal to or lower than their expenses. Those whose income exceeded their expenses had higher death depression compared with those whose income was lower than their expenses.

**Table 2.** Death Distress Scale (DDS) scores according to women’s descriptive characteristics, earthquake experiences, fertility characteristics, and change in fertility intentions Table 2 long description.

	Death distress scale total score (mean ± sd)	Death anxiety (mean ± sd)	Death depression (mean ± sd)	Death obsession (mean ± sd)
**Education**				
Primary education	25.71 ± 7.81	7.48 ± 3.08	10.88 ± 3.88	7.35 ± 3.83
High school	25.40 ± 7.37	7.84 ± 3.12	10.89 ± 3.47	6.68 ± 3.14
University and further	25.93 ± 7.28	7.11 ± 3.17	11.34 ± 3.52	7.47 ± 3.57
Test Değeri	.128	1.346	.496	1.584
*p*-value	.880	.262	.609	.207
Working**				
Working	25.96 ± 7.10	6.93 ± 3.21	11.69 ± 3.34	7.34 ± 3.87
Not working	25.56 ± 7.58	7.69 ± 3.08	10.82 ± 3.67	7.05 ± 3.39
Test Değeri	.382	−1.772	1.752	.590
*p*-value	.703	.077	.081	.584
Income*				
Income-less than expenses	26.08 ± 8.68	8.34 ± 3.30	10.50 ± 3.94	7.24 ± 3.63
Income equals expenses	25.70 ± 7.33	7.61 ± 2.83	10.93 ± 3.38	7.16 ± 3.71
Income exceeds expenses	24.78 ± 5.02	5.81 ± 2.81	12.17 ± 3.29	6.79 ± 2.70
Test Değeri	.570	13.318	4.166	.324
*p*-value	.566	.000***	.016***	.724
**Damage to houses lived in due to earthquake**				
No damage occurred	24.39 ± 8.17	6.63 ± 2.98	10.84 ± 3.99	6.92 ± 3.34
Minor damage occurred	25.79 ± 7.02	7.83 ± 2.84	11.03 ± 3.80	6.94 ± 3.39
Moderate damage occurred	26.02 ± 7.85	8.15 ± 3.54	10.91 ± 3.35	6.96 ± 3.76
High level of damage occurred	26.28 ± 7.19	7.29 ± 3.21	11.26 ± 3.17	7.72 ± 3.62
Test Değeri	.810	2.952	.172	.872
*p*-value	.489	**.033**	.915	.456
**Situation under rubble in an earthquake**				
Yes	26.28 ± 6.67	6.00 ± 2.81	12.83 ± 2.90	7.44 ± 3.94
No	25.61 ± 7.52	7.61 ± 3.12	10.90 ± 3.63	7.10 ± 3.48
Test Değeri	.367	−2.133	2.213	.407
*p*-value	.714	**.030**	**.028**	.684
**Injury in earthquake**				
Yes	26.73 ± 7.97	7.65 ± 3.65	11.24 ± 3.18	7.84 ± 3.71
No	25.44 ± 7.35	7.49 ± 3.02	10.98 ± 3.69	6.98 ± 3.45
Test Değeri	1.113	.301	.476	1.577
*p*-value	.267	.765	.634	.116
**Experiencing loss due to earthquake**				
Yes	25.72 ± 7.86	7.74 ± 3.14	10.78 ± 3.53	7.19 ± 3.55
No	25.60 ± 7.18	7.35 ± 3.11	11.19 ± 3.67	7.06 ± 3.47
Test Değeri	.134	1.085	−.962	.310
*p*-value	.894	.279	.337	.757
Intended to have a child before the earthquake (*n* = 105)**				
No change (or sooner) (*n* = 44)	24.55 ± 7.25	6.66 ± 2.86	11.14 ± 3.55	6.75 ± 3.52
Later or not at all (*n* = 61)	29.15 ± 7.91	8.61 ± 3.23	11.77 ± 3.04	8.77 ± 3.65
Test Değeri	−3.046	−3.198	−.983	−2.840
*p*-value	**.003**	**.002**	.328	**.005**
**Total sample (*n* = 296)**	25.65 ± 7.46	7.51 ± 3.12	11.02 ± 3.61	7.12 ± 3.50

*One-way ANOVA **İndependent *T*-test ***Post-hoc Tukey.

Death anxiety differed significantly according to the condition of the house damaged by the earthquake (*p* < .05). Those who stated that their house was moderately damaged after the earthquake had higher levels of death anxiety than those who stated that their house was undamaged. Furthermore, those who were trapped under debris during the earthquake had lower death anxiety and higher death depression than those who were not (*p* < .05) (Table 2).

Relationships between fertility characteristics, fertility intention, and death distress are presented below: Accordingly, there was a significant difference in death distress and death anxiety between women who experienced a change in fertility intentions and those who did not (*p* < .05). Compared with women who did not experience a change in fertility intentions, those who did had higher levels of death distress, death anxiety, and death obsession (Table 2).

Table 3 shows the relationship between participants’ death distress and age, number of pregnancies, and number of children. Accordingly, a weak negative correlation was observed between age and death anxiety (*r* = −.199, *p* < .01). The number of pregnancies was weakly negatively correlated with death anxiety (*r* = −.195, *p* < .01) and weakly positively correlated with death depression (*r* = .151, *p* < .05). There were a weak negative correlation between the number of children and death anxiety (*r* = −.231, *p* < .01) and a weak positive correlation between the number of children and death depression (*r* = .168, *p* < .01). In addition, death obsession showed a weak positive correlation with death anxiety (*r* = .304, *p* <.01) and a moderate positive correlation with death depression (*r* = .479, *p* < .01).

**Table 3.** Examining the relationship between women’s Death Distress Scale (DDS) scores, age, number of pregnancies, and number of children (*n* = 296) Table 3 long description.

Variables	1	2	3	4	5	6	7
1-Age	1.000	–	–	–	–	–	–
2-Number of pregnancies	.621*	1.000	–	–	–	–	–
3-Number of children	.641*	.866*	1.000	–	–	–	–
4-Death distress scale total score	−.055	.004	−.011	1.000	–	–	–
5-Death anxiety	−.199*	−.195*	−.231*	.564*	1.000	–	–
6-Death depression	.058	.151*	.168*	.712*	.027	1.000	–
7-Death obsession	−.001	-.011	−.025	.824*	.304*	.479*	1.000

Spearman’s Correlation *p < .01.

## Discussion

The researchers’ aims are fourfold. Determination of death distress levels in women after the earthquake was carried out first. Accordingly, mean death distress level among the participants was found to be 25.65 ± 7.46. Birni *et al*. conducted a study with the survivors of the February 6, 2023 earthquake and found that the death distress levels of those directly affected by the earthquake (27.22 ± 7.01) were higher compared to those of individuals who had a loved one or second-degree relative affected by the earthquake (26.62 ± 6.90) and individuals unaffected by the earthquake (25.31 ± 9.72), and this difference was statistically significant. The death-distress levels of earthquake survivors reported by Bırni et al. are consistent with the findings of the present study.

Generally, death distress has a multidimensional structure including death anxiety, death depression, and death obsession (Dadfar and Lester, 2020). Death depression is the most prominent feature of death distress and is defined as the grief experienced by an individual in relation to death (Alkış, 2023). This study identified bereavement-related depression as the most important factor associated with death-related distress among women. The results of another study showed that death depression is highest within 7–12 months after the loss of a loved one (Armas-Arráez *et al*., 2023). The high level of death depression in the present study can be explained by the study being conducted within the first year after the earthquake. Death anxiety, conceptualized as a key component of death distress, encompasses the fears, negative emotions, and thoughts that individuals experience in relation to their own mortality and the potential loss of significant others (Abdel-Khalek, 2004; Menzies and Menzies, 2023; Menzies *et al*., 2024). In the present study, participants’ death anxiety levels were moderate and constituted the second most significant component, after death depression. This finding is consistent with the growing body of evidence indicating that earthquake survivors commonly experience elevated anxiety symptoms; studies following the 2023 Kahramanmaraş earthquakes found that more than 51% of adult survivors screened positive for psychological distress, with women identified as a particularly vulnerable group (Çıtak and Dadandı, 2024). Furthermore, research conducted specifically on death anxiety among earthquake victims in Turkey has confirmed that this construct is significantly elevated in post-disaster populations (Cenat, 2020; Akturk and Yenigun, 2024; Gümüş Şekerci *et al*., 2024). The third component of death distress identified in this sample was death obsession, which involves recurring thoughts, intrusive images, and rumination focused on one’s own death or the death of significant others (Yıldırım and Güler, 2021). Death obsession levels were moderate among the participants. Major disasters, such as earthquakes, remind people of their vulnerability and the fleeting nature of life. This can increase thoughts about death and lead people to consider their own deaths and those of their loved ones.

In this study, a negative correlation was found between age and death anxiety, indicating a decrease in death anxiety with increasing age. This finding is consistent with longitudinal evidence demonstrating that death anxiety tends to decline with advancing age, as older individuals may develop greater psychological adaptation and acceptance of mortality over time (Guo *et al*., 2024). These differences may be attributed to older adults developing a fear of living because of physical problems and social isolation. As the fear of living becomes more dominant than the fear of death, death anxiety decreases among older adults. On the contrary, it is argued that young individuals experience more death anxiety compared to elderly individuals because they have more to do in life and have not yet achieved their goals (Gökha, 2010). The findings obtained in the present study support this view.

In the present study, individuals with higher income levels had lower levels of death anxiety and higher levels of death depression. As indicated by Hallegatte *et al*. (2017), disasters can disproportionately affect low-income individuals, leaving them homeless. However, the study’s findings suggest that lower levels of death anxiety among individuals with higher incomes may be attributed to their residence in safer housing and to their greater resilience to natural disasters such as earthquakes. High income levels can provide an advantage in coping with disasters by offering more financial resources and strength, leading individuals to feel more secure and reduce their death anxiety (Huang *et al*., 2021).

Death anxiety among women decreases as the numbers of pregnancies and children increase. Becoming a parent can reduce death anxiety in individuals through factors such as finding the meaning and purpose of life, ensuring the continuation of life, and seeing themselves contributing to a greater purpose (Nomaguchi and Milkie, 2020). Women whose houses were moderately damaged experienced higher death anxiety than those whose houses were not damaged. This indicates that the fear and anxiety experienced during the earthquake tend to increase death anxiety among individuals whose homes are damaged. Additionally, greater economic and social losses may contribute to increased death anxiety. Therefore, understanding the negative effects of the earthquake on individuals’ mental health and providing support can be crucial.

In the present study, women trapped under debris had lower death anxiety but higher death depression. The women trapped under the debris may have felt a sense of survival. This, together with the awareness of the dangerous situation, can create a sense of inner strength and resilience that supports survival, leading to a reduction in anxiety about death. However, women trapped under debris may experience depression and grief. This may be linked to losses and trauma and lead to a higher incidence of death-related depression. This process of depression and grief leads to a decrease in death anxiety, but it may also lead to an increase in death depression (Yıldırım and Güler, 2021; Kalyoncu *et al*., 2023).

Fertility intention is one of the most robust predictors of couples’ fertility behavior and is influenced by various factors. The literature emphasizes that fertility decisions are filled with uncertainty. These uncertainties are considered the reason for the mismatch between fertility intentions and actual fertility. Recent disasters, such as the coronavirus disease 2019 (COVID-19) pandemic and earthquakes, contribute to this uncertainty and lead to postponement of fertility intentions (Malicka *et al*., 2021). The results obtained in the present study support this view. While 36.5% of women expressed the intention to have a child within the next 3 years before the earthquake, more than half of these women reported changes to their fertility intentions after the earthquake, including postponing them or completely giving up on them. Studies examining the impact of Covid-19 pandemic, which is considered a biological disaster, on fertility intentions have found that approximately one-third of women postponed their fertility intentions or expressed a desire to have fewer children (Lindberg *et al*., 2020). In a study covering Italy, France, Germany, Spain, and the United Kingdom, 38%–58% of individuals reported postponing their fertility intentions due to the Covid-19 pandemic (Luppi *et al*., 2020). As a result of the earthquake, families often face economic difficulties and may have fewer financial resources. This may affect married women’s intentions to have children, since economic uncertainty and reduced resources can increase the financial burden of having children, thereby reducing fertility intentions.

According to the results of the present study, individuals with changes in fertility intentions had higher levels of death distress, death anxiety, and death obsession compared with those without such changes. Losses and traumatic experiences caused by earthquakes can affect the grief and loss processes of women. Changes in fertility intentions can be perceived as a need to mourn losses and complete the mourning process. This process of grief and loss may lead to an increase in death anxiety and death obsession. After an earthquake, women often experience psychological stress and trauma. Changes in fertility intentions may reflect stress and trauma experienced by individuals, leading to an increase in death anxiety and obsession.

## Conclusions

The present study found a moderate level of death distress among women. Psychological symptoms, such as death anxiety, depression, and obsession, which constitute a significant component of traumatic experiences following an earthquake, are essential for assessing the emotional and psychological needs of individuals and for providing appropriate support services. Individuals experiencing distress related to death may need psychological support. These symptoms may negatively affect activities of daily living and lead to long-term psychological health problems. Death distress has emerged as a crucial component in the diagnosis of psychological trauma among trauma-exposed patients. Measurement of these symptoms is therefore essential for identifying appropriate treatment and rehabilitation programs.

The results also showed that more than half of the women who intended to give birth before the earthquake either postponed or completely abandoned their plans. Changes in fertility intentions are an important source of data for the development and implementation of family planning policies and programs. Based on these data, governments and local authorities can provide family planning services tailored to the needs of post-earthquake communities.

Changes in fertility intentions are closely related to fertility rates, but they are also highly variable due to external factors. According to the results of the present study, individuals with changes in fertility intentions had higher levels of death distress, death anxiety, and death obsession compared to those without such changes. Therefore, our study contributes a new perspective and additional information to literature.

## Limitations

In interpreting the study data, certain important limitations should be considered. First, a control group not exposed to the earthquake could not be included in this study. For this reason, the sample in which the effects of the earthquake were examined could not be compared to a control group to eliminate confounding effects of other variables that may be related to mental health. Another limitation is that the data were collected online, which means that the population consisted of individuals who had internet access, owned a computer or smartphone, and knew how to use these technologies. Therefore, the results obtained can be generalized to the participants and are valid for the time period during which the research was conducted.

In the present study, the causal relationship between death distress and fertility intention could not be confirmed. Future studies should evaluate and confirm this relationship in more detail.

## Data Availability

The data underlying this article in the original language will be shared by the corresponding author upon reasonable request.

## Figure 1. Long description

A flowchart illustrating the impact of an earthquake on individuals’ intentions to have a child within the next three years. The first decision point asks if the individual intended to have a child before the earthquake. If the answer is no, the next question is whether the earthquake has changed this intention, with possible responses being no, intending to have a child sooner or more, intending to have a child later, or deciding never to have a child. If the initial answer is yes, the same follow-up question is asked with the same possible responses. The flowchart uses rectangles for decision points and branching paths to indicate different outcomes based on the individual’s response.

Navigate back to Figure 1..

## Table 1. Long description

The table presents data on women’s demographics, earthquake experience, and fertility characteristics. It includes information on age, number of pregnancies, number of children, education level, working status, income, damage to houses due to the earthquake, situation under rubble, injury during the earthquake, experiencing loss, family planning methods used before and after the earthquake, and fertility intentions. The table has 296 entries and covers various statistics and percentages related to these categories.

Navigate back to Table 1..

## Table 2. Long description

The table presents data on death distress scale scores, including death anxiety, death depression, and death obsession, across different characteristics and experiences of women. It includes categories such as education level, working status, income, damage to houses due to an earthquake, situation under rubble in an earthquake, injury in an earthquake, experiencing loss due to an earthquake, and intended fertility before the earthquake. The table has 19 rows and 17 columns, with each row representing a specific characteristic or experience and each column representing a different aspect of the death distress scale. Notable trends include variations in death distress scores based on income levels, damage to houses, and experiences during the earthquake.

Navigate back to Table 2..

## Table 3. Long description

The table presents data on the relationship between women’s death distress scale scores, age, number of pregnancies, and number of children. It includes seven variables: age, number of pregnancies, number of children, death distress scale total score, death anxiety, death depression, and death obsession. The table has seven rows and seven columns, with correlation coefficients and significance values indicated. Notable trends include a weak negative correlation between age and death anxiety, and a weak positive correlation between the number of pregnancies and death depression. The number of children shows a weak negative correlation with death anxiety and a weak positive correlation with death depression. Death obsession is weakly positively correlated with death anxiety and moderately positively correlated with death depression.

Navigate back to Table 3..

## References

[ref1] Abdel-Khalek AM (2004) Death anxiety, death depression, and death obsession: A general factor of death distress is evident: A reply. Psychological Reports 94(3), 1212–1214. 10.2466/pr0.94.3c.1212-1214 15362394

[ref2] AFAD (2023) About Earthquakes in Kahramanmaras – *36* . Available at https://www.afad.gov.tr/kahramanmarasta-meydana-gelen-depremler-hk-36 (accessed 8 August 2023).

[ref3] Akturk U and Yenigun H (2024) Examination of death anxiety of earthquake victims in Türkiye. Journal of Social and Analytical Health 4(1), 1–6. 10.5281/zenodo.10976044

[ref4] Alkış MS (2023) *Examining the Reliability and Validity of the Revised Death Anxiety Scale and Death-Related Depression Scale in Nurses* (Master’s Thesis, Istanbul Sabahattin Zaim University). (in Turkish).

[ref5] Armas-Arráez MM , Padilla GM , Fernández-Mateos LM and Sánchez-Cabaco A (2023) Death distress and religiosity among Spanish patients diagnosed with depression and anxiety. OMEGA - Journal of Death and Dying 92(1), 495–510. 10.1177/00302228231186369 37365885

[ref6] Ayllón S (2019) Job insecurity and fertility in Europe. Review of Economics of the Household 17(4), 1321–1347. 10.1007/s11150-019-09450-5

[ref7] Bırni G , Deniz ME , Karaağaç ZG , Erişen Y , Kaya Y and Satıcı SA (2023) Rebuilding wellbeing: Understanding the role of self-criticism, anger rumination, and death distress after the February 6, 2023, Türkiye earthquake. Death Studies 48(5), 511–521. 10.1080/07481187.2023.2241401 37534943

[ref8] Brée S , Bourguignon M and Eggerickx T (2016) La fécondité en Europe occidentale durant l’Entre-deux-guerres. Quels effets des crises sur les comportements démographiques? Annales De Démographie Historique 132(2), 41. 10.3917/ADH.132.0041

[ref9] Cénat JM , McIntee SE and Blais-Rochette C (2020) Symptoms of posttraumatic stress disorder, depression, anxiety and other mental health problems following the 2010 earthquake in Haiti: A systematic review and meta-analysis. Journal of Affective Disorders 273, 55–85. 10.1016/j.jad.2020.04.046 32421623

[ref43] Centers for Disease Control and Prevention (CDC) (2021) Reproductive Health in Emergency Preparedness and Response. Atlanta: Centers for Disease Control and Prevention. https://www.cdc.gov/reproductivehealth/emergency/index.html

[ref10] Çıtak Ş. and Dadandı İ. (2024) The effect of earthquake exposure on PTSD symptoms is mediated by intrusive rumination and moderated by gender. BMC Public Health 24, 2294. 10.1186/s12889-024-19736-8 39180034 PMC11342482

[ref11] Dadfar M and Lester D (2020) Death distress constructs: A preliminary empirical examination of the Farsi form in nurses: A brief note. Nursing Open 7(4), 1026–1031. 10.1002/nop2.484 32587721 PMC7308705

[ref12] Eurostat (2020) Fertility indicators [demo_find]. Available at https://ec.europa.eu/eurostat/data/database (accessed 5 June 2026).

[ref41] Faul F , Erdfelder E , Buchner A and Lang AG (2009) Statistical power analyses using G*Power 3.1: tests for correlation and regression analyses. Behavior Research Methods 41(4), 1149–1160. 10.3758/BRM.41.4.1149 19897823

[ref13] Göka E (2010) Dying: The Psychology of Death and Those Left Behind. İstanbul: Timaş Press.

[ref14] Grace K (2017) Considering climate in studies of fertility and reproductive health in poor countries. Nature Climate Change 7, 479–485. 10.1038/nclimate3318 PMC6009846 29937922

[ref16] Gümüş Şekerci Y , Ayvazoğlu G and Çekiç M (2024) The effect of depression and hopelessness level on death anxiety in earthquake survivor students one year after the earthquake. OMEGA - Journal of Death and Dying. Advance online publication. 10.1177/00302228241295417 39431303

[ref15] Guo J , Wister A , Mitchell B and Li S (2024) Number of chronic conditions and death anxiety among older adults in rural China: a longitudinal study in Anhui province. Journal of Aging & Health 37(10), 632–643. 10.1177/08982643241289516. (Original work published 2025). 39361775

[ref17] Hallegatte S , Vogt-Schilb A , Bangalore M and Rozenberg J (2017) Unbreakable: Building the Resilience of the Poor in the Face of Natural Disasters. World Bank. 10.1596/978-1-4648-1003-9

[ref18] Huang S , Du H and Qu C (2021) Emotional responses to mortality salience: Behavioral and ERPs evidence. PloS One 16(3), e0248699. 10.1371/journal.pone.0248699 33730033 PMC7968674

[ref19] Kalyoncu N , Aydoğdu A , Polat I , Karabulut SN , Keskin M and Özkan M (2023) The wounded minds of wounded bodies: evaluation of consultation-liaison psychiatry nursing services in a university hospital after the February 6th, Kahramanmaras Earthquakes. Abant Journal of Health Sciences and Technologies 3(3), 13–22. (in Turkish).

[ref20] Kartol A , Üztemur S and Yaşar P (2024) Development and validation of the earthquake obsession scale. Death Studies 49(3), 219–227. 10.1080/07481187.2024.2317177 38372351

[ref21] Kristensen P , Weisæth L and Heir T (2012) Bereavement and mental health after sudden and violent losses: A review. Psychiatry-Interpersonal and Biological Processes 75(1), 76–97. 10.1521/psyc.2012.75.1.76 22397543

[ref22] Lau BH , Huo R , Wang K , Shi L , Li R , Mu S , Peng H , Wang Y , Chen X , Ng EH and Chan CH (2018) Intention of having a second child among infertile and fertile women attending outpatient gynecology clinics in three major cities in China: A cross-sectional study. Human Reproduction Open 2018(4), hoy014. 10.1093/hropen/hoy014 30895255 PMC6276692

[ref23] Lee DS , Batyra E , Castro A and Wilde J (2023) Human fertility after a disaster: A systematic literature review. Proceedings of the Royal Society B: Biological Sciences 290(1998), 20230211. 10.1098/rspb.2023.0211 PMC10170212 37161332

[ref24] Leech N , Barrett K and Morgan GA (2007) SPSS for Intermediate Statistics: Use and Interpretation. 3rd edn. Mahwah, New Jersey (NJ): Routledge. 10.4324/9781410616739

[ref26] Lindberg LD , VandeVusse A , Mueller J and Kirstein M (2020) *Early Impacts of the COVID-19 Pandemic: Findings from the 2020* Guttmacher Survey of Reproductive Health Experiences. Guttmacher Institute. Available at https://www.guttmacher.org/sites/default/files/report_pdf/early-impacts-covid-19-pandemic-findings-2020-guttmacher-survey-reproductive-health.pdf (accessed 5 June 2026).

[ref27] Luppi F , Arpino B and Rosina A (2020) The impact of COVID-19 on fertility plans in Italy, Germany, France, Spain, and the United Kingdom. Demographic Research 43, 1399–1412. 10.4054/DemRes.2020.43.47

[ref28] Malicka I , Mynarska M and Świderska J (2021) Perceived consequences of the COVID-19 pandemic and childbearing intentions in Poland. Journal of Family Research 33(3), 674–702. 10.20377/jfr-666

[ref25] Marteleto LJ , Padilla SK , Dondero M and Sereno LGF (2023) Fertility intentions during the COVID-19 pandemic: An analysis of individual- and municipality-level determinants. Population and Development Review 50(S1), 213–242. 10.1111/padr.12561 39145111 PMC11323109

[ref30] Menzies RE , McMullen K , Riotto GD , Iliescu S , Petrovic B and Remfrey M (2024) From dread to disorder: A meta-analysis of the impact of death anxiety on mental illness symptoms. Clinical Psychology Review 113, 102490. 10.1016/j.cpr.2024.102490 39208495

[ref29] Menzies RE and Menzies RG (2023) Death anxiety and mental health: Requiem for a dreamer. Journal of Behavior Therapy & Experimental Psychiatry 78, 101807. 10.1016/j.jbtep.2022.101807 36435549

[ref42] Mohammadzadeh A , Ashouri A , Vahedi M and Asgharipour N (2018) Death distress dimensions: Death anxiety, death depression, and death obsession. Journal of Fundamentals of Mental Health 20(6), 341–348. 10.22038/jfmh.2018.11754

[ref31] Mussino E , Gabrielli G , Ortensi LE and Strozza S (2021) Fertility intentions within a 3-year time frame: A comparison between migrant and native Italian women. Journal of International Migration and Integration 24(SUPPL. 1), 233–260. 10.1007/s12134-020-00800-2

[ref32] Muttarak R (2021) Demographic perspectives in research on global environmental change. Population Studies 75, 77–104. 10.1080/00324728.2021.1988684 34902278

[ref33] Nakajima Ş. (2012) Post-earthqake psychology. Okmeydanı Medical Journal 28(2), 150–155. 10.5222/otd.supp2.2012.150. (in Turkish).

[ref34] Nomaguchi K and Milkie MA (2020) Parenthood and well-being: A decade in review. Journal of Marriage and the Family 82(1), 198–223. 10.1111/jomf.12646 32606480 PMC7326370

[ref35] Özmen B (2023) Why were the Kahramanmaraş Earthquakes so Devastating? Available at https://www.aa.com.tr/tr/analiz/gorus-kahramanmaras-depremleri-neden-bu-kadar-yikici-oldu/2817774 (in Turkish) (accessed 5 June 2026).

[ref37] Şeker BD and Akman E (2014) Emotional, cognitive and behavioral reactions after Van earthquake: Examination of police sample. Uludağ University Faculty of Arts and Sciences Journal of Social Sciences 15(27), 215–231. 10.21550/sosbilder.269510. (in Turkish).

[ref36] Somefun O , Banougnin BH and Smith-Greenaway E (2024) The relationships between drought exposure, fertility preferences, and contraceptive behaviors: A multicountry study. Studies in Family Planning 55(1), 5–21. 10.1111/sifp.12258 38414154

[ref38] Yıldırım M and Güler A (2021) Positivity explains how COVID-19 perceived risk increases death distress and reduces happiness. Personality & Individual Differences 168, 110347. 10.1016/j.paid.2020.110347 32843780 PMC7439822

[ref39] Yıldız B and Akkoyun AZ (2023) Psychiatric support after earthquake. Izmir Katip Çelebi University Faculty of Health Sciences Journal 8(2), 817–820. https://dergipark.org.tr/en/pub/ikcusbfd/issue/78150/1267011

[ref40] Zotti ME , Williams AM , Robertson M , Horney J and Hsia J (2013) Post-disaster reproductive health outcomes. Maternal and Child Health Journal 17(5), 783–796. 10.1007/s10995-012-1068-x 22752348 PMC4540175

